# Real-World Effectiveness of Frontline Treatments Among Patients with Chronic Lymphocytic Leukemia: Results from ConcertAI

**DOI:** 10.3390/cancers17050799

**Published:** 2025-02-26

**Authors:** Lindsey E. Roeker, John M. Burke, Joanna M. Rhodes, Nnadozie Emechebe, Dureshahwar Jawaid, Beenish S. Manzoor, Christopher E. Jensen, Lindsay Ryland, Yangyang Liu, Steve E. Marx, Wendy Sinai, Jordan Roser, Mazyar Shadman

**Affiliations:** 1 CLL Program, Leukemia Service, Division of Hematologic Oncology, Memorial Sloan Kettering Cancer Center, New York, NY 10065, USA; 2 Rocky Mountain Cancer Centers, Aurora, CO 80012, USA; 3 Rutgers Cancer Institute of New Jersey, New Brunswick, NJ 08901, USA; 4 AbbVie, Inc., North Chicago, IL 60064, USA; 5 Lineberger Comprehensive Cancer Center, University of North Carolina at Chapel Hill, Chapel Hill, NC 27517, USA; 6 Fred Hutchinson Cancer Research Center, Seattle, WA 98109, USA

**Keywords:** chronic lymphocytic leukemia, real-world outcomes, treatment effectiveness

## Abstract

**Background:** The long-term follow-up of clinical trials of novel first-line (1L) therapies for chronic lymphocytic leukemia (CLL) demonstrates 6–10-year progression-free survival. We describe the effectiveness of 1L CLL treatments in real-world settings, with an emphasis on the important real-world outcome of time to next treatment or death (TTNT-D). **Methods:** This retrospective, observational study utilized de-identified electronic health records from the ConcertAI RWD360™ database with linked administrative open claims. Adults with CLL who initiated an approved 1L CLL therapy (June 2019–March 2023) were included. Duration of therapy (DoT), TTNT-D, and overall survival were assessed. **Results:** At 1L, 39.8% of 1843 patients received first-generation covalent Bruton tyrosine kinase inhibitors (cBTKis), 23.0% second-generation cBTKis, 12.4% venetoclax-obinutuzumab (VenO), 7.4% chemotherapy/chemoimmunotherapy (CT/CIT), and 17.4% anti-CD20 monotherapy. Median (range) follow-up in months was 24.9 (13.1–36.6) for first-generation cBTKis, 13.4 (7.3–21.7) for second-generation cBTKis, 16.0 (8.4–27.8) for VenO, 21.8 (11.2–32.7) for CT/CIT, and 19.7 (10.0–33.4) for anti-CD20 monotherapy. Median (range) DoT was 11.5 (4.2–25.0) and 8.6 (3.0–16.1), 9.1 (5.9–12.2), 5.6 (3.2–5.8), and 1.6 (1.6–4.5) months for first- and second-generation cBTKis, VenO, CT/CIT, and anti-CD20 monotherapy, respectively. Regarding TTNT-D, at 2 years’ follow-up, 69.1%, 82.5%, 86.3%, 79.1%, and 53.0% of patients treated with first- and second-generation cBTKis, VenO, CT/CIT, and anti-CD20 monotherapy, respectively, had not initiated subsequent treatment or experienced death. **Conclusions:** TTNT-D is an important real-world outcome in CLL. Our findings demonstrated the utility of time-limited VenO, with potentially more time off treatment, relative to continuous 1L cBTKi therapies.

## Simple Summary

Advances in therapies for patients with chronic lymphocytic leukemia (CLL) have led to prolonged progression-free survival as evidenced by recent clinical trial data, while real-world data from clinical practice settings are emerging. We describe the effectiveness of front-line CLL treatments in adult patients with CLL in real-world settings. Results demonstrated that venetoclax-based and chemotherapy/immunotherapy treatments were utilized as expected fixed-duration treatments, while covalent Bruton tyrosine kinase inhibitors (cBTKis) had longer durations of therapy because they are administered as continuous therapy. More recent novel therapies of second-generation cBTKis and venetoclax-based regimens led to prolonged time to receipt of next treatment or death, demonstrating benefits relative to older regimens of first-generation cBTKis, chemotherapy/immunotherapy, and anti-CD20 monotherapy. Coupled with the potential benefits that venetoclax-based regimens may offer with time off treatment as a time-limited therapy, these findings should be considered as part of shared decision-making with the goal of optimizing patient outcomes.

## 1. Introduction

The treatment landscape in chronic lymphocytic leukemia (CLL) is continuously evolving to improve patient outcomes, and the selection of first-line (1L) treatment is increasingly important [1,2]. Until 2014, treatment options predominantly included cytotoxic chemotherapy (CT) agents utilized as monotherapy or various combinations, including combinations with immunotherapies, such as anti-CD20 agents [1,3].

In more recent years, targeted therapies have been introduced that provide CT-free treatment options for patients with CLL. These therapies, including covalent Bruton tyrosine kinase inhibitors (cBTKis) and B cell lymphoma-2 (BCL-2) inhibitors, have improved clinical outcomes, including progression-free survival and toxicity profiles, which are more manageable relative to CT/chemoimmunotherapy (CIT) [1,4,5,6,7,8,9,10]. Ibrutinib was the first-in-class cBTKi (i.e., first-generation) and is a continuous treatment option, with long-term follow-up studies demonstrating a well-known toxicity profile including atrial fibrillation, hypertension, and ventricular arrhythmias [11]. More recently, second-generation cBTKis, including acalabrutinib and zanubrutinib, were approved, with more favorable toxicity profiles [11,12,13]. Venetoclax, a BCL-2 inhibitor, is also approved for treatment of front-line and relapsed/refractory CLL, either as monotherapy or in combination with anti-CD20 agents rituximab or, more recently (May 2019), obinutuzumab (VenO) [9,10,14,15,16,17]. These combination therapies induce deep responses that allow for a fixed-duration treatment [16,17,18]. Venetoclax has a differing mechanism of action and safety profile relative to cBTKi with potential for tumor lysis syndrome, which is not seen with cBTKi therapy [9,10]. Real-world evidence of venetoclax for patients with CLL demonstrate effectiveness, durability, and an acceptable safety profile [19].

Given the lack of head-to-head trials comparing novel targeted agent front-line treatment options, assessing all available treatment options across a suite of key endpoints in a real-world setting has become increasingly important. While there is a growing body of real-world evidence on the effectiveness of treatments for CLL, most data come from academic institutions and may not be representative of patients seen in routine clinical practice. While maximized overall survival (OS) is the ultimate endpoint to target in patients with CLL, reporting of this endpoint is often lacking for both trials and real-world studies given the long time to maturation for OS data [20,21,22]. Time to next treatment or death (TTNT-D) has emerged as a key endpoint for evaluating treatment effectiveness in real-world practice settings, serving as a proxy for progression-free survival to inform treatment decision-making [23,24,25]. For fixed-duration and continuous regimens alike, TTNT-D shows time to active symptoms after progression. Given that patients may receive subsequent therapy for reasons beyond disease progression (i.e., toxicity, patient preference, insurance coverage), TTNT-D reflects not only treatment efficacy and disease control but also patient-centric aspects of compliance and tolerability [23,24,25,26,27,28,29,30]. However, real-world studies have utilized different endpoint definitions and rules for the derivation of line(s) of therapy (LoT), limiting the utility of data describing the TTNT-D endpoint. Differences in reported TTNT-D findings between studies highlight the importance of the derivation of the LoT algorithm, with a selection window and rules for advancing the LoT that consider dosing/prescribing patterns for all treatments analyzed [19,31,32,33]. The variability in TTNT-D definitions speaks to the importance of rigorous methodology in defining the endpoint and LoT, which would make the assessment of TTNT-D more reliable and consistent across studies. This descriptive study aimed to analyze and describe the real-world effectiveness of 1L CLL treatments in predominantly community settings, with an emphasis on TTNT-D defined in a clear and consistent manner.

## 2. Methods

### 2.1. Data Source and Patients

This retrospective, observational study utilized de-identified electronic health records (EHRs) from the ConcertAI RWD360™ database with linked administrative open claims from January 2010 to June 2023. De-identified EHRs are derived from patient clinical charts in oncology centers across the US from patients treated at various academic and community centers. RWD360™ consists of rigorous, standardized, clinical, patient-level, longitudinal data from multiple EHRs that are representative of the US population. The EHRs are linked to administrative claims data that are related to the eligible patient population to enrich the data source. The ConcertAI RWD360™ database was utilized in several other retrospective studies within oncology [34,35,36,37,38].

Patients aged ≥18 years who were diagnosed with CLL or small lymphocytic lymphoma between January 2010 and March 2023 (based on International Classification of Diseases [ICD]-10 codes), initiated an approved 1L CLL therapy between June 2019 and March 2023, had ≥1 month of activity in the ConcertAI database prior to diagnosis, and had two structured visits post-diagnosis were included in this study. The index date was defined as the start date of the first therapy received on or after the initial CLL diagnosis. Patients were treated in academic and community settings. Patients were excluded from this study if they had a diagnosis of any other malignancy at any time prior to or on the index date based on the presence of ≥1 record with ICD-10 codes, had evidence of clinical trial participation at any time prior to or on the index date to minimize risk that the regimen was administered as part of a clinical trial, or had evidence of mortality prior to or on the index date. ICD-9 diagnosis codes have been mapped into ICD-10 diagnosis codes in the ConcertAI database; thus, only ICD-10 codes were utilized in the analysis.

### 2.2. Cohorts

Patients were divided into 1 of 5 treatment groups (first-generation cBTKis, second-generation cBTKis, VenO, CT/CIT, anti-CD20 monotherapy) based on the 1L regimen received as determined by the derived LoT.

### 2.3. Outcomes Assessed

Real-world duration of therapy (DoT), TTNT-D, and OS were assessed and expressed in months. DoT was defined as the number of days from the start to the end of treatment in each LoT for all patients and among a subset of patients with at least 18 months of follow-up. TTNT-D was defined as the time in days from the start of the first LoT to the start of the next LoT or death. OS was defined as the time in days from the date of treatment initiation to the date of death plus 1 day. For TTNT-D and OS, patients without these events during the assessment windows were censored at the earlier of either the end of patient activity in the database or the end of the study period. The proportion of patients receiving each treatment regimen at 1L and subsequent lines (among those who received subsequent treatment) was determined. Duration of follow-up was derived by taking the difference between the start of 1L and the occurrence of death, the end of patient activity in the database, the end of data availability, or the end of the study period, whichever came first.

### 2.4. Analysis

This study was descriptive in nature. Data were summarized as mean and standard deviation (SD), median values and ranges for continuous variables, and frequency and proportions for categorical variables. Time to event analyses (i.e., TTNT-D and OS) were carried out using Kaplan–Meier (KM) methodology, and KM estimates were generated to evaluate TTNT-D and real-world OS at 12 and 24 months.

The LoT were derived up to the third line of therapy with clinician input based on the following algorithm:

- All medications indicated for CLL at any LoT were included to derive the LoT. Orally administered medications were required to be ordered (EHR) or dispensed (claims) while intravenously (IV) administered medications were required to be administered irrespective of the source. The inclusion of open claims data was used to enrich the creation of additional LoT per patient and to augment the capture of oral drug refills, which may be inadequately captured in EHR databases.
- The 1L regimen was defined as all medications ordered/dispensed (orals) or administered (IV/intramuscular) within 35 days of the index date. The first date of a qualifying medication in the regimen served as the date of the first LoT.
- The new LoT was defined based on the following events: the addition of a new therapeutic agent to combination regimens any time after the 35-day window; the addition of a new therapeutic agent to monotherapies any time after the 180-day window; the switching of an entire regimen; or the re-initiation of the same regimen after a gap of >90 consecutive days for medications administered IV only [24].
  ○ A distinction was made between monotherapy and combination regimens in the addition of an agent rule to minimize the risk of misclassifying the late addition of anti-CD20 agents or venetoclax to a cBTKi or chemotherapy agent as a new LoT based on clinician input and use in the published literature [24]. Furthermore, the re-initiation rule was limited to IV administered medications because refills of oral drugs are not consistently captured in EHR databases.

In the sensitivity analysis, the addition of another therapeutic agent any time after the 35-day window advanced the LoT for all regimens, while other characteristics of the algorithm remained the same. All analyses were carried out using SAS v9.4.

## 3. Results

### 3.1. Baseline Characteristics

**Figure 1:**
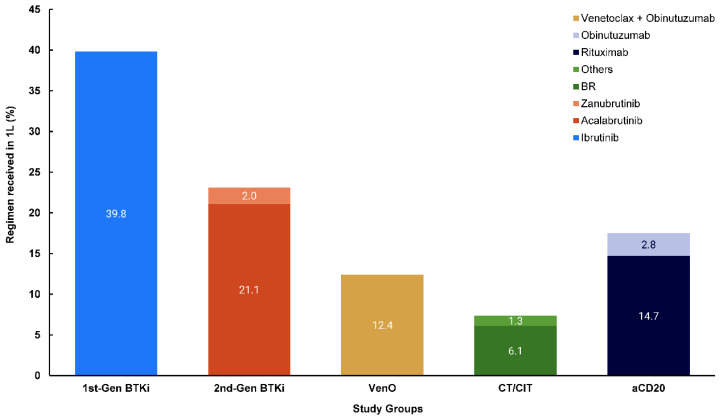
Distribution of 1L regimens by treatment groups. Others included fludarabine, cyclophosphamide, rituximab, and other CT/CIT agents. 1L, first-line; aCD20, anti-CD20; BR, bendamustine plus rituximab; cBTKi, covalent Bruton tyrosine kinase inhibitor; CT/CIT, chemotherapy/chemoimmunotherapy; gen, generation; VenO, venetoclax plus obinutuzumab.

A total of 1843 patients were included in this analysis (Table 1), of which 1423 and 383 were treated in community versus academic centers, respectively. At 1L of therapy, 39.8% (n = 733) were treated with first-generation cBTKis, followed by 23.0% (n = 424) receiving second-generation cBTKis, 12.4% (n = 229) VenO, 7.4% (n = 136) CT/CIT, and 17.4% (n = 321) anti-CD20 (Figure 1). Baseline characteristics were unweighted; thus, there were differences observed in baseline characteristics detailed in Table 1. For example, mean (SD) age for patients varied across treatment groups, ranging from 65.7 (9.9) years for VenO to 71.4 (10.7) years for anti-CD20, with patients who received cBTKis or anti-CD20 regimens generally older than patients who received VenO or CT/CIT (Table 1). Median time from CLL diagnosis to initiation of 1L therapy was 13.2 and 16.8 months for patients receiving first- and second-generation cBTKis, respectively, 11.8 months for VenO, 2.6 months for CT/CIT, and 2.1 months for anti-CD20.

The median (interquartile range) follow-up time in months was 24.9 (13.1–36.6) for first-generation cBTKis, 13.4 (7.3–21.7) for second-generation cBTKis, 16.0 (8.4–27.8) for VenO, 21.8 (11.2–32.7) for CT/CIT, and 19.7 (10.0–33.4) for anti-CD20.

### 3.2. DoT

The median DoT for first- and second-generation cBTKis was 11.5 (4.2–25.0) and 8.6 (3.0–16.1) months, respectively; among patients with ≥18 months of follow-up, the median DoT for first- and second-generation cBTKis was 20.7 and 19.7 months, respectively, indicating that cBTKis are being used as continuous therapies.

The DoT was 9.1 (5.9–12.2) months for VenO and 5.6 (3.2–5.8) months for CT/CIT, which is standard for CT/CIT regimens (Table 2); similar findings were observed when selecting patients with ≥18 months of follow-up time, with the VenO regimen DoT (11.6 [7.9–13.2] months) confirming use of venetoclax as a fixed-duration therapy as per the prescribing label [41].

Median (range) DoT was shortest among patients who received anti-CD20 at 1.6 (1.6–4.5) months overall and 1.8 (1.6–5.5) months among patients with ≥18 months of follow-up. Generally, DoT was similar for patients treated in academic (n = 383) and community-based (n = 1423) settings across most 1L regimens, and numerically shorter in patients receiving first- or second-generation cBTKis in the academic relative to community setting (Table 2).

### 3.3. TTNT-D

**Figure 2:**
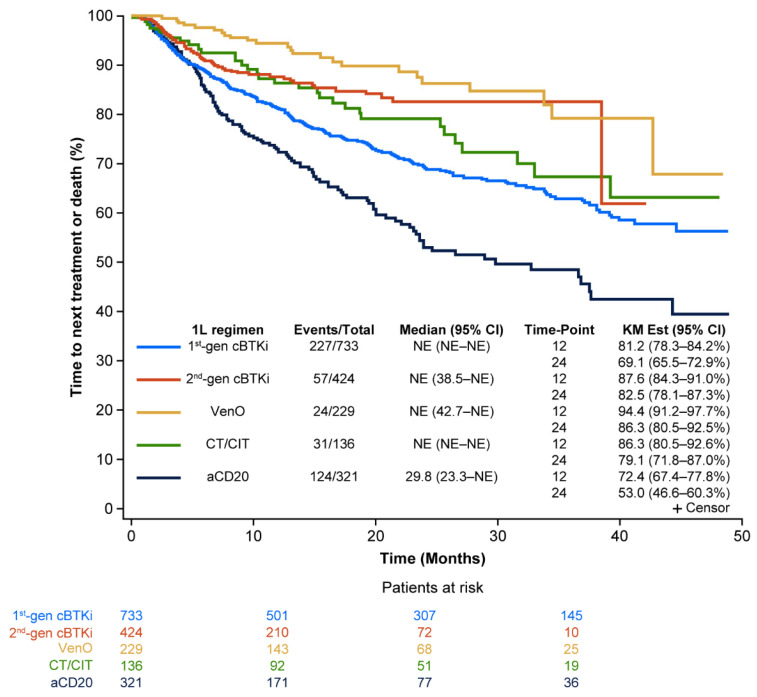
Real-world TTNT-D for CLL treatments in 1L. 1L, first-line; aCD20, anti-CD20; cBTKi, covalent Bruton tyrosine kinase inhibitor; CI, confidence interval; CLL, chronic lymphocytic leukemia; CT/CIT, chemotherapy/chemoimmunotherapy; gen, generation; NE, not estimable; KM, Kaplan–Meier; TTNT-D, time to next treatment or death; VenO, venetoclax plus obinutuzumab.

The median (95% confidence interval [CI]) TTNT-D was not reached across patients who received first- or second-generation cBTKis, VenO, or CT/CIT regimens and was 29.8 (23.3–not estimable) months in patients treated with anti-CD20. Based on KM estimates at 24 months, 69.1% (95% CI: 65.5–72.9) of patients treated with 1L first-generation cBTKis had not received a subsequent LoT or experienced death, while 82.5% (95% CI: 78.1–87.3) for second-generation cBTKis, 86.3% (95% CI: 80.5–92.6) for VenO, 79.1% (95% CI: 71.8–87.0) for CT/CIT, and 53.0% (95% CI: 46.6–60.3) for anti-CD20 had not initiated subsequent therapy or experienced death (Figure 2).

### 3.4. OS

**Figure 3:**
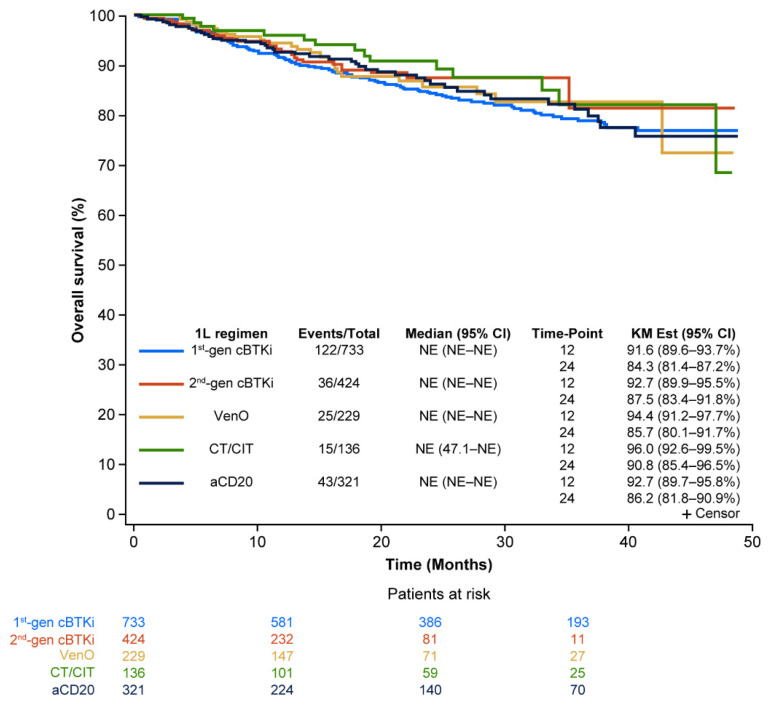
Real-world OS for CLL treatments in 1L. 1L, first-line; aCD20, anti-CD20; cBTKi, covalent Bruton tyrosine kinase inhibitor; CI, confidence interval; CLL, chronic lymphocytic leukemia; CT/CIT, chemotherapy/chemoimmunotherapy; gen, generation; KM, Kaplan–Meier; NE, not estimable; OS, overall survival; VenO, venetoclax plus obinutuzumab.

The median OS was not reached across all treatment groups in the primary analysis. At 24 months, 84.3% (95% CI: 81.4–87.2) for first-generation cBTKis, 87.5% (95% CI: 83.4–91.8) for second-generation cBTKis, 85.7% (95% CI: 80.1–91.7) for VenO, 90.8% (95% CI: 85.4–96.5) for CT/CIT, and 86.2% (95% CI: 81.8–90.9) for anti-CD20 had not experienced death (Figure 3).

**Figure 4:**
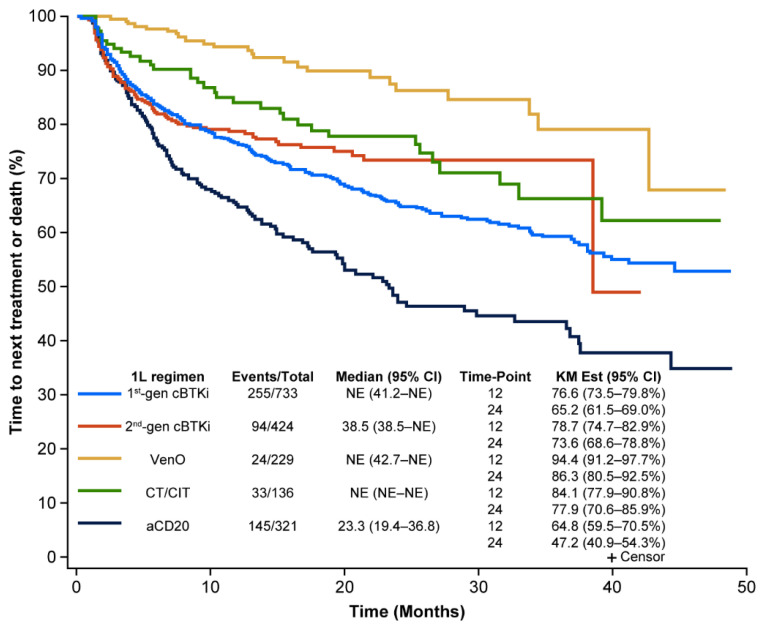
Real-world TTNT for CLL treatments in 1L: sensitivity analysis. 1L, first-line; aCD20, anti-CD20; cBTKi, covalent Bruton tyrosine kinase inhibitor; CI, confidence interval; CLL, chronic lymphocytic leukemia; CT/CIT, chemotherapy/chemoimmunotherapy; gen, generation; KM, Kaplan–Meier; NE, not estimable; TTNT-D, time to next treatment or death; VenO, venetoclax plus obinutuzumab.

**Figure 5:**
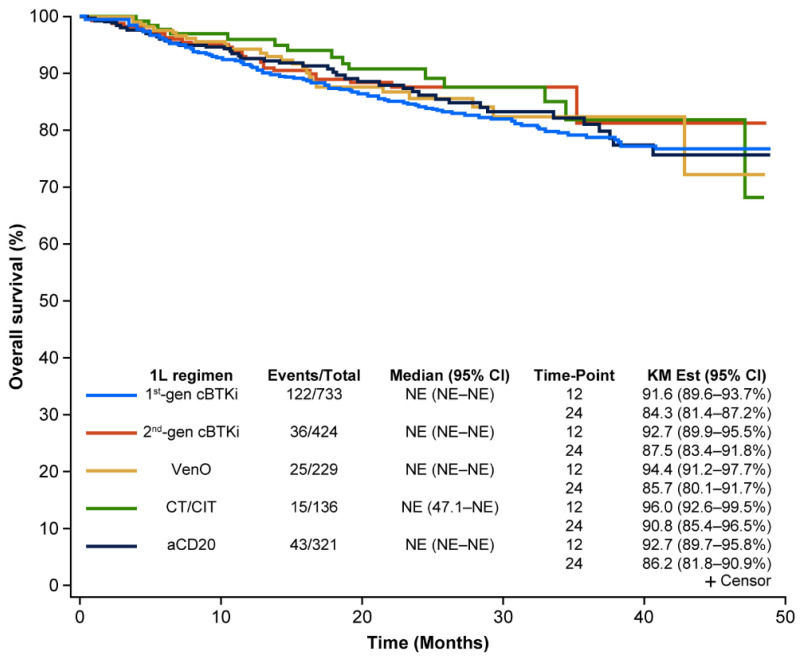
Real-world OS for CLL treatments in 1L: sensitivity analysis. 1L, first-line; aCD20, anti-CD20; cBTKi, covalent Bruton tyrosine kinase inhibitor; CI, confidence interval; CLL, chronic lymphocytic leukemia; CT/CIT, chemotherapy/chemoimmunotherapy; gen, generation; KM, Kaplan–Meier; NE, not estimable; OS, overall survival; VenO, venetoclax plus obinutuzumab.

In the sensitivity analysis, where the addition of any agent any time after the 35-day window advanced the LoT for all regimens, median TTNT-D and OS showed similar trends to the primary analysis; however, as would be expected, the percentage of patients who had not switched therapies or experienced death was lower overall across most treatment groups relative to the primary analysis. Additionally, the median TTNT-D was truncated in the sensitivity analysis for second-generation cBTKis and anti-CD20 (due to a higher number of discontinuations or additions of a new therapeutic agent that advanced the LoT recorded with a shorter time window), highlighting the importance of careful consideration of events and parameters that advance the LoT that is appropriate for all treatments included within an analysis (Figure 4 and Figure 5).

### 3.5. Subsequent Treatments

**Figure 6:**
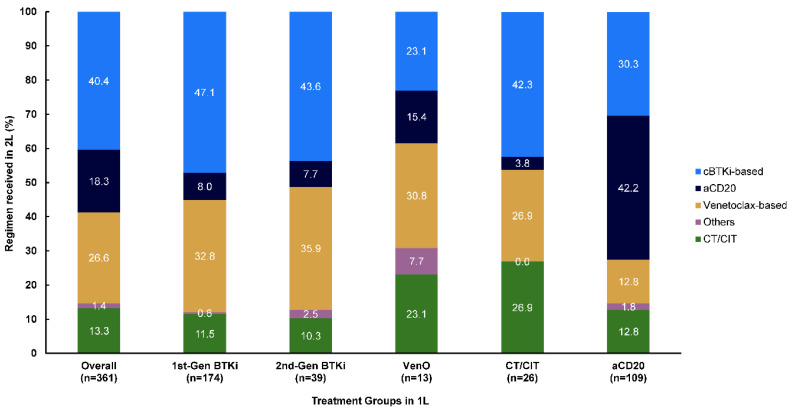
Subsequent treatments received at 2L stratified by 1L treatment groups. Other treatments included duvelisib (n = 1) and lenalidomide (n = 4). 1L, first-line; 2L, second-line; aCD20, anti-CD20; cBTKi, covalent Bruton tyrosine kinase inhibitor; CT/CIT, chemotherapy/chemoimmunotherapy; gen, generation; VenO, venetoclax plus obinutuzumab.

Overall, 361 patients moved on to a next LoT (Figure 6). The less frequent subset to move to a next LoT was patients who received 1L VenO (13/361, 3.6%), of whom most moved on to either other cBTKi-based (3/13, 23.1%), venetoclax-based (4/13, 30.8%), or CT/CIT-based (3/13, 23.1%) treatments (Figure 6). Of patients receiving 1L first- and second-generation cBTKis, 48.2% (174/361) and 10.8% (39/361), respectively, moved to a next LoT; among these patients, 47.1% (82/174) and 43.6% (17/39), respectively, moved on to another cBTKi-based treatment, while 32.8% (57/174) and 35.9% (14/39), respectively, moved on to a venetoclax-based regimen. More specifically, of patients who received 1L first- or second-generation cBTKis and moved on to another cBTKi-based treatment in second-line, 32.2% (56/174) and 3.8% (3/39) switched to acalabrutinib-based, 1.7% (3/174) and 15.4% (6/39) switched to ibrutinib-based, and 3.2% (23/174) and 20.5% (8/39) switched to zanubrutinib-based cBTKis, respectively (Supplementary Table S1).

## 4. Discussion

The treatment landscape for CLL is evolving, with an increasing focus on optimal 1L therapy selection, as there is no prospective clinical trial comparing the currently approved regimens [2]. In this real-world study, we assessed unweighted DoT, TTNT-D, and OS across five different therapeutic regimens received in 1L CLL settings. Importantly, this study assessed these findings descriptively without adjustments for differences in baseline characteristics, thus providing a look at real-world performance in clinical settings where the selection of treatment regimen is often influenced by patient characteristics; for example, age varied across treatment groups in our study, which can be an important consideration in treatment selection. Findings from this study may aid in treatment decision-making regarding 1L therapy selection in clinical practice settings.

Regarding DoT, our findings demonstrate that the real-world DoT for VenO and CT/CIT is aligned with the expected fixed treatment durations per the respective protocols. While EHR data may have inherent limitations in measuring DoT for oral medications, as the data reflect only physician orders and not pharmacy fills, we also assessed data for patients with ≥18 months of follow-up time and found similar trends. We further demonstrated that VenO and CT/CIT are utilized as fixed-duration therapies, with median real-world DoT of 11.6 and 5.6 months, respectively, in patients with longer follow-up times. As may be expected with treat-to-progression continuous therapy protocols, DoT was longest among patients treated with 1L first- and second-generation cBTKis; this was particularly prominent among patients with ≥18 months of follow-up. These findings demonstrate consistency in the real-world setting with prescribing information and demonstrate the rigor of the results reported.

TTNT-D is an important endpoint in broader oncology and is especially relevant in CLL. As noted by healthcare providers and researchers alike, TTNT-D remains a meaningful endpoint as it not only uniquely reflects treatment efficacy and disease control, but also patient-centric aspects of compliance and tolerability to a regimen [23,24,25,26,27,28,29,30]. In CLL specifically, TTNT has been used as a meaningful measure for progression-free survival [24], though changes in therapy may be driven by factors other than disease progression. TTNT-D shows time to active symptoms requiring further treatment after progression, becoming an important consideration for clinical decision-making. Additionally, markers of progression differ between clinicians and between clinical practices, making it difficult to capture reliably in real-world datasets, further highlighting the utility of TTNT-D as an important endpoint. In our study, although the median TTNT-D was not reached for any regimen, given the relatively short follow-up in all groups, the analysis of TTNT-D at 2 years after initiation of 1L therapy demonstrated that fixed-duration VenO had the highest rate of patients yet to initiate a next LoT or experience death (86%), followed by first-generation cBTKis (69%), second-generation cBTKis (83%), CT/CIT (79%), and anti-CD20 (53%). These results suggest that the more recent novel therapies (second-generation cBTKis and VenO) are achieving a better balance of durable remissions, manageable toxicities, and prolonged progression-free survival as compared with older therapies.

The median TTNT-D for VenO observed in this study was similar to estimates reported from many other real-world studies assessing TTNT-D [32,33,42], and stands in contrast to the findings of Chanan-Khan and colleagues, who reported that the median TTNT was significantly longest for patients treated with cBTKis (53.9 months) and shortest for patients treated with venetoclax-based therapies (7.7 months) in 1L [31]. This discrepancy may be explained by possible differences in the capture window time frame and rules that were used for advancing the LoT in the derivation of the LoT algorithm. As LoT are not provided directly within claims data, careful consideration of the events and parameters that advance the LoT for analysis is needed, especially in a complex and evolving treatment landscape, to help avoid misclassification of 1L and subsequent treatments. In our study, the sensitivity analysis findings were consistent with the primary analysis for VenO and inconsistent for cBTKis, with truncated TTNT-D using a 35-day window capture; this finding further highlights that dosing/prescribing patterns must be considered when deriving LoT algorithms and the importance of analyzing data with rigorous statistical methods. The assessment of DoT and TTNT-D in real-world datasets should be meticulous as these results have implications on treatment decision-making and, ultimately, patient outcomes.

Real-world evidence on sequencing, following the discontinuation or completion of therapy, post-1L therapy is still emerging as an important consideration for clinical practice, as switching or discontinuing therapy carries implications for healthcare providers, healthcare systems, payers, and patients alike. While premature discontinuation may indicate intolerability of a regimen, moving to a subsequent line or switching therapy increases healthcare resource use and costs, clinician management time, possible financial toxicity, and inconveniences for patients [43,44,45,46]. That said, our study illustrates that patients receiving first-generation cBTKis were the largest proportion to move on to a next LoT (47% to another cBTKi and 33% to a venetoclax-based regimen), followed by anti-CD20 agents, CT/CIT, and second-generation cBTKi, with the VenO group as the smallest subset to move on to a next LoT. Not only are our results consistent with those of Ghosh et al., but they further suggest durability of response with VenO while offering patients time off treatment, and validate the fixed-duration recommendation for venetoclax-based regimens [42]. These findings are notable, as time off treatment affords patients a myriad of benefits, including reduced exposure to side effects, less financial toxicity, fewer disturbances and more conveniences in their daily routine, and fewer mental health/psychosocial effects [46,47,48,49,50]. Taken together, these cumulative benefits of a fixed-duration therapy, in contrast to continuous therapy, should be considered in shared decision-making, with the goal of optimizing patient outcomes.

Notably, in the current study, 17.5% of patients received anti-CD20 as 1L therapy. The high use of this therapy in 1L is significant, given that patients who initiate this regimen as monotherapy in 1L typically have poor outcomes [32,51]. In this study, the majority of patients were classified as true monotherapy users, with a small number on a combination regimen. While patients receiving anti-CD20 alone may be older and more frail, consideration of a novel agent or a combination of anti-CD20 with a novel agent as opposed to anti-CD20 monotherapy may be better suited for optimizing patient outcomes [52].

The findings from this analysis are among the first to report evidence across a large mixed community and academic dataset on the real-world effectiveness of venetoclax-based and other more novel therapies. Interestingly, DoT in the community setting was generally slightly longer than in the academic setting across most treatment groups; this was particularly prominent for cBTKis. An exception was with anti-CD20 monotherapy, with which there was a numerically longer DoT in the academic versus community setting.

### Strengths and Limitations

A strength of this study is that it assesses real-world data from oncology practices, including detailed information combining EHR, chart reviews, lab data, and integrated claims data. This study uses aggregated EHR data collected from predominantly community oncology practices, which include clinical data and demographics, highlighting 1L treatment patterns and outcomes in community clinical practice settings. Additionally, our median TTNT-D results for VenO were similar to estimates reported from many other real-word studies [32,33,42], suggesting that the methodology applied was both statistically rigorous and appropriate. Finally, this is one of the first studies to descriptively evaluate both first- and second-generation cBTKis versus a venetoclax-based regimen; while the follow-up may be relatively short, these early data are still meaningful as second-generation cBTKis become used more frequently in clinical practice. A longer duration of follow-up may be needed to fully assess differences in outcomes across treatments. This study was descriptive in nature and as such does not account for differences in baseline characteristics that may meaningfully impact outcomes; however, these unadjusted data allow us to understand the performance of these regimens in patients for whom each regimen is being selected.

Due to the limitation of EHR data records, even with abstraction, there are inherent limitations to this study. Records in the inpatient setting are only captured if the data is available in the EHR in the abstraction; this may impact the accuracy of oral dosage of all treatments in the inpatient setting. Furthermore, physician notes regarding the planned or unplanned nature of treatment interruptions may be limited, and patients can only be followed as long as they continue to receive treatment at the oncology clinic that contributes data to ConcertAI. Reasons for treatment discontinuation (i.e., toxicity or disease progression) or change are not available within the database. Additionally, observing DoT with EHRs may not be fully representative of actual medication intake as it measures physician orders and not pharmacy fills; however, we attempted to mitigate this in part by integrating administrative claims data in the analysis.

## 5. Conclusions

The findings of this study demonstrate beneficial outcomes for patients with CLL treated in real-world clinical practice. Novel therapies of VenO and second-generation cBTKis achieve prolonged TTNT-D relative to older regimens of first-generation cBTKis, CT/CIT, and anti-CD20 alone. While TTNT-D is an increasingly important real-world outcome, especially in CLL, the methodology to study this outcome must be scientifically rigorous and fit for purpose, as demonstrated in this study, to reliably make treatment decisions that advance patient care and outcomes.

## Figures and Tables

**Table 1:** Baseline Characteristics.

Characteristics	1st-Gen cBTKi (n = 733)	2nd-Gen cBTKi (n = 424)	VenO (n = 229)	CT/CIT (n = 136)	aCD20 (n = 321)
**Age at 1L,** Mean (SD) [median]	70.1 (10.6) [70.0]	71.2 (10.0) [72.0]	65.7 (9.9) [66.0]	66.9 (11.0) [67.0]	71.4 (10.7) [73.0]
**Gender, n (%)** Male	430 (58.7)	249 (58.7)	139 (60.7)	83 (61.0)	142 (44.2)
**Time from CLL diagnosis to 1L in months,** Mean (SD) [median]	25.9 (30.0) [13.2]	27.6 (30.9) [16.8]	22.4 (28.5) [11.8]	18.5 (27.2) [2.6]	17.1 (27.0) [2.1]
**Absolute lymphocyte count (10^3^/μL),** Mean (SD) [median]	56.0 (66.3) [31.5]	63.3 (73.2) [36.8]	67.5 (93.9) [35.2]	49.6 (108) [6.6]	22.2 (49.7) [2.9]
**Quan [39,40] CCI score,** Mean (SD) [median]	1.9 (1.5) [2.0]	2.1 (1.7) [2.0]	2.2 (1.5) [2.0]	2.1 (1.3) [2.0]	2.3 (1.3) [2.0]
**ECOG performance status,** n (%)					
Missing	388 (52.9)	219 (51.7)	108 (47.2)	65 (47.8)	139 (43.3)
Grade 0	177 (24.1)	102 (24.1)	65 (28.4)	38 (27.9)	96 (29.9)
Grade 1	136 (18.6)	86 (20.3)	46 (20.1)	25 (18.4)	60 (18.7)
Grade 2	22 (3.0)	17 (4.0)	10 (4.4)	7 (5.1)	19 (5.9)
Grade 3	8 (1.1)	0 (0.0)	0 (0.0)	1 (0.7)	6 (1.9)
Grade 4	2 (0.3)	0 (0.0)	0 (0.0)	0 (0.0)	1 (0.3)

1L, first-line; aCD20, anti-CD20; cBTKi, covalent Bruton tyrosine kinase inhibitor; CCI, Charlson Comorbidity Index; CLL, chronic lymphocytic leukemia; CT/CIT, chemotherapy/chemoimmunotherapy; ECOG, Eastern Cooperative Oncology Group; gen, generation; SD, standard deviation; VenO, venetoclax plus obinutuzumab.

**Table 2:** DoT by 1L treatment for all patients and among patients with at least 18 months of follow-up.

Overall DoT in Months, Median (IQR)	Overall (n = 1843)	With ≥18 Months of Follow-Up Time (n = 971)
First-generation cBTKis	11.5 (4.2–25.0)	20.7 (7.8–31.5)
Second-generation cBTKis	8.6 (3.0–16.1)	19.7 (13.1–24.8)
VenO	9.1 (5.9–12.2)	11.6 (7.9–13.2)
CT/CIT	5.6 (3.2–5.8)	5.6 (3.7–6.0)
Anti-CD20	1.6 (1.6–4.5)	1.8 (1.6–5.5)
**Academic Setting DoT in Months,** Median (IQR)	**Overall** **(n = 383)**	**With ≥18 Months of Follow-Up Time** **(n = 211)**
Overall	6.6 (2.3–13.1)	10.4 (3.9–23.4)
First-generation cBTKis	10.6 (3.6–25.2)	20.3 (8.9–31.0)
Second-generation cBTKis	7.1 (1.9–12.2)	17.1 (1.9–23.7)
VenO	9.8 (6.2–12.2)	10.4 (6.2–12.3)
CT/CIT	5.5 (3.8–5.7)	5.5 (3.7–5.7)
Anti-CD20	1.8 (1.6–4.6)	2.5 (1.6–4.8)
**Community Setting DoT in Months,** Median (IQR)	**Overall** **(n = 1423)**	**With ≥18 Months of Follow-Up Time** **(n = 760)**
Overall	7.0 (2.3–15.8)	13.1 (4.4–24.5)
First-generation cBTKis	11.5 (4.4–24.5)	20.7 (7.4–31.3)
Second-generation cBTKis	9.0 (3.2–16.7)	20.1 (13.2–25.0)
VenO	8.9 (5.8–12.0)	11.7 (8.3–13.7)
CT/CIT	5.6 (3.0–6.0)	5.6 (3.8–6.2)
Anti-CD20	1.6 (1.6–4.5)	1.6 (1.6–5.6)

1L, first-line; cBTKi, covalent Bruton tyrosine kinase inhibitor; CT/CIT, chemotherapy/chemoimmunotherapy; DoT, duration of therapy; IQR, interquartile range; VenO, venetoclax plus obinutuzumab.

## Data Availability

The data analyzed in this study are subject to Health Insurance Portability and Accountability Act privacy restrictions and are not publicly available. De-identified data could be made available by the corresponding author upon reasonable request. AbbVie is committed to responsible data sharing regarding the clinical trials we sponsor. This includes access to anonymized, individual, and trial-level data (analysis data sets), as well as other information (e.g., protocols, clinical study reports, or analysis plans), as long as the trials are not part of an ongoing or planned regulatory submission. This includes requests for clinical trial data for unlicensed products and indications. These clinical trial data can be requested by any qualified researchers who engage in rigorous, independent, scientific research, and will be provided following review and approval of a research proposal, statistical analysis plan, and execution of a data sharing agreement. Data requests can be submitted at any time after approval in the US and Europe and after acceptance of this manuscript for publication. The data will be accessible for 12 months, with possible extensions considered. For more information on the process or to submit a request, visit the following link: https://vivli.org/ourmember/abbvie/ then select “Home”.

## Supplementary Materials

The following supporting information can be downloaded at: https://www.mdpi.com/article/10.3390/cancers17050799/s1, Table S1. Next regimen by study group.
