# Supplementary material for: Real-World Effectiveness of Frontline Treatments Among Patients with Chronic Lymphocytic Leukemia: Results from ConcertAI
Source: Cancers (Basel). 2025 Feb 26;17(5):799. doi: 10.3390/cancers17050799 (PMC11899398; doi:10.3390/cancers17050799)
Supplement: Supplementary file 1 [file cancers-17-00799-s001.zip › cancers-3367923-supplementary.pdf]

Supplementary Materials

Table S1. Next regimen by study group

**Next Regimen (2L)**	**Study Group by 1L Treatment Regimen**					
**n (%)**	**1st-Gen cBTKi (n=174)**	**2nd-Gen cBTKi (n=39)**	**VenO (n=13)**	**CT/CIT (n=26)**	**aCD20 (n=109)**	**Total (N=361)**
**Acalabrutinib-based**	56 (32.2)	3 (7.7)	1 (7.7)	6 (23.1)	14 (12.8)	80 (22.2)
**CT/CIT**	20 (11.5)	4 (10.3)	3 (23.1)	7 (26.9)	14 (12.8)	48 (13.3)
**Ibrutinib-based**	3 (1.7)	6 (15.4)	0 (0.0)	3 (11.5)	13 (11.9)	25 (6.9)
**Others[a]**	1 (0.6)	1 (2.6)	1 (7.7)	0 (0.0)	2 (1.8)	5 (1.4)
**Venetoclax-based**	57 (32.8)	14 (35.9)	4 (30.8)	7 (26.9)	14 (12.8)	96 (26.6)
**Zanubrutinib-based**	23 (13.2)	8 (20.5)	2 (15.4)	2 (7.7)	6 (5.5)	41 (11.4)
**aCD20**	14 (8.1)	3 (7.7)	2 (15.4)	1 (3.9)	46 (42.2)	66 (18.3)
**Total**	174 (48.2)	39 (10.8)	13 (3.6)	26 (7.2)	109 (30.2)	361 (100.0)

[a]Other treatments included duvelisib (n=1) and lenalidomide (n=4).

1L, first-line; 2L, second-line; aCD20, anti-CD20; cBTKi, covalent Bruton tyrosine kinase inhibitor; CT/CIT, chemotherapy/chemoimmunotherapy; gen, generation; VenO, venetoclax plus obinutuzumab.
